# Carbon risk and green transition: evidence from China

**DOI:** 10.3389/fpubh.2023.1346145

**Published:** 2024-01-30

**Authors:** Junfu Li, Yanxiang Xie, Xiang Gao, Qian Wei

**Affiliations:** ^1^Business School, Nankai University, Tianjin, China; ^2^Research Center of Finance, Shanghai Business School, Shanghai, China; ^3^School of International Economics and Trade, Guangxi University of Foreign Languages, Nanning, China

**Keywords:** carbon risk, green transition, path mechanisms, heterogeneity analysis, China

## Abstract

Carbon risk may have potential influences on the green transition of enterprises. This paper thoroughly investigates the effect and mechanism of carbon risk on the transition towards sustainability. We use quantitative regression models and a panel of Chinese manufactural listed companies from 2011-2020. There is strong evidence manifesting that the effect of carbon risk on corporate green transition is positive and statistically significant. The green transition is marked by the overall encouragement of exploratory, exploitable, autonomous, and collaborative green innovation. The mechanism test indicates that the enhancement of internal R&D transformation and the pressure of external stakeholders are two fundamental pathways by which carbon risk influences the green transition. Additional examination reveals that the beneficial impact is particularly noticeable for companies that have limited capital intensity, minimal governmental assistance, reduced financial limitations, and are state-owned enterprises. These results are robust to resolve the problem of endogeneity by means of instrumental variables, Heckman two-step, placebo test, propensity score matching and difference-in-difference ways. Against the background of carbon neutrality, it is of great significance to examine the relationship between carbon risk and corporate green transition. The conclusion complements the knowledge of carbon risk and green transition, as well as provides theoretical insights and practical enlightenment for the green transition of manufacturing enterprises in emerging economies.

## 1 Introduction

Green development has become a hot issue under discussion of the international community (1). Green transition has become an inevitable choice for sustainable development of emerging market countries (2). As a major component of the economy, manufacturing enterprises serve as a pivotal player in the green transition (3). This paper empirically investigates how enterprise’s carbon risk influences the green transition of Chinese manufacturing listed companies from 2011 to 2020 based on 2,163 samples and 12,939 enterprise-year observations. Considering that the entry into force of China’s environmental protection tax law in 2018 is an exogenous event, we test for changes in the relationship between carbon risk and green transition. On this basis, we further identify the underlying paths mechanism of carbon risk affecting the green transition. To make sure the robustness of the result, we carried out decomposition mechanism test and heterogeneity analysis.

As a potential environmental disadvantage, carbon risk signifies the risk of uncertainty linked to climate variation or fossil energy excessive use (4). This uncertain risk related to current and future environmental policies and regulations may have an enormous influence on the company’s business activities and financial performance by means of supererogatory costs of compliance or emission trading mechanisms. Latest studies have tried to investigate the effect of carbon risk on different levels of investment or financing of enterprises. According to Bolton and Kacperczyk (5), the presence of carbon risk has a beneficial effect on the performance of stocks. Certain studies indicate that the release of carbon contributes to the vulnerabilities faced by businesses, including elevated credit risk (6), increased loan expenses (7, 8) and additional financial burdens (9). The presence of carbon risk may cause enterprises to decrease their investments and dividends, resulting in a certain degree of reduction in investment efficiency (10, 11). Furthermore, the company’s valuation will be adversely impacted by carbon risk, leading to an increase in capital cost and causing deviation from the optimal leverage ratio (12, 13). However, some studies have found that the intensification of carbon risk prompts the reinforcement of ecological governance, thereby incentivizes businesses to engage in eco-friendly technological advancements. This, in turn, helps offset a portion or even more of the expenses incurred due to environmental regulations. Consequently, the compensatory effect of such innovation can drive the transition toward sustainability for enterprises (14). Relevant studies indicate that implementing moderate environmental regulations can reduce the overall pollution degree of society by affecting the environmental behavior of enterprises (15). This can result in a multi-win condition of regional environmental, economic, and social benefits (16). Stringent regulations on the environment can incentivize enterprises to engage in innovative activities such as environmental technology innovation and ecological innovation. This can enhance the green overall productivity of enterprises by means of promoting environmental research and development (R&D) (17–19) as well as venture-backed innovation (20). Conversely, the escalation of environmental regulations due to increasing carbon risks exacerbates the business risks and associated management expenses of companies (10). When faced with higher carbon risk and more stringent environmental policy, both production cost and emission reduction cost will experience a significant rise. This will limit the innovation resources available, hinder the green innovation efforts of businesses, decelerate the progress of transitioning toward sustainability, and introduce higher levels of uncertainty in future business activities (9). Based on the perspective of stakeholders, higher carbon risks will bring greater pressure from stakeholders, and enterprises will actively respond to stakeholder pressure and adopt green practices to meet stakeholders’ demands for environmental concerns (21, 22). To date until now, there are no consistent conclusions about whether and how carbon risks affect the green transition. What is more, the current research sample primarily consists of companies that provide data on carbon emissions. The predicament of self-selection and small-sample bias may be encountered by these research designs.

Carbon emissions have become a hot issue and have attracted extensive attention from academia and industry. China, being the biggest developing nation and the largest industrialized country globally, currently holds the record for having the highest carbon emissions within a single country. Consequently, in the context of carbon neutrality, China is confronting with immense pressure to cut down carbon emissions, which leads to challenges in transitioning toward a greener future. As a key component of China’s economy, manufacturing industry accounts for a large proportion of China’s carbon emissions. Hence, the achievement of carbon neutrality heavily relies on the successful green transitioning of manufacturing enterprises in China. How does carbon risk affect the green transition of enterprises is an important issue worth studying. The government employs stringent regulation measures and direct tax policy to facilitate the green transition of firms. China implemented its environmental protection tax law in the year 2018. The implementation of this tax policy encourages firms to undertake environmentally friendly measures and decrease their carbon footprint. Hence, the public policy in China offers an exceptional situation for investigating how carbon risks affect the transition of enterprises toward sustainability. At the same time, as a representative of emerging market countries, China’s theory and practice of green transition of manufacturing enterprises provide important reference significance for other emerging market countries.

Hence, three marginal contributions are made in this study. Firstly, we expand the related knowledge on carbon risk and transition toward sustainability at the micro firm-level, both in terms of theoretical connotation and empirical evidence. A quasi-natural experimental environment is designed to preferably recognize the causal association between carbon risk and green transition behavior with finer granular firm-level sample data. In addition, the paper also provides initial proof for the effect of carbon risk in emerging markets and the impact of public policies in enterprise behavior. Secondly, we proposed a new measurement method of green transition, used the number of green patent applications as the proxy variable of green transition, as well as identified four novel types of green transition: exploitative, exploratory, autonomous and cooperative green transition for the first time. Thirdly, we opened the black box of the relationship between carbon risk and green transition, and revealed for the first time that internal R&D transformation and external stakeholder pressure are underlying path mechanisms through which carbon risk affects corporate green transition.

The study is organized as follows. Section2 exposes theoretical analysis and hypothesis. Section 3 describes methods. We discuss results in section 4. Section 5 reveals paths mechanism and heterogeneity tests. Finally, the conclusion is drawn.

## 2 Theoretical analysis and hypothesis development

### 2.1 Carbon risk and green transition

Carbon risk generally refers to the influence of society’s sustainable transition to a low-carbon economy paradigm on firm value due to policy, legal, technology, market and reputation changes (23). In fact, carbon risk is a transition risk. Many existing studies on carbon risk primarily examine how carbon risk affects various aspects of investment and financing in businesses. For instance, implementing stringent carbon supervision policy will increase the uncertainty of future cash flow and lead to financial default of enterprises (7); the carbon risks lead to the increasing of financial predicament risks, which propel firms to lower financial lever (Nguyen and Phan 2020); the carbon risk will increase the income uncertainty of enterprises, significantly increase the bank loan cost of enterprises, and reduce the dividend payment ratio (8, 10). However, institutional and stakeholder theories suggest different views on this. Within institutional theory, it is argued that “stakeholder engagement” is important for companies to establish social legitimacy (24). As posited by stakeholder theory, stakeholder pressures result in significant motivation for organizations to adopt various environmental practices (25–27). The rising carbon risk has aroused the concern of stakeholders on environmental issues and brought about greater stakeholder pressure, which can force enterprises to introspect their own shortcomings in carbon emission reduction, overcome organizational inertia, and turn external pressure into the driving force for companies to actively seek green product and process innovation (28). To date until now, there is still no consistent conclusion between carbon risk and green transition. “Porter Hypothesis” holds that the rise of carbon risk will result in stronger environmental supervision, and more stringent environmental constraint may urge companies to carry out green innovation, resulting innovation compensation effect can propel the green transition of enterprises (14). The opposite viewpoint argues that rising carbon risk will increase the environmental regulation compliance cost of enterprises, squeeze out R&D resources and inhibit their innovation activities, thus hindering the green transition of enterprises (9).

According to the “Porter Hypothesis,” the rising of carbon risk will result in stronger environmental supervision, which can propel firms to engage in green innovation and facilitate the transition toward sustainability. To a certain degree, the entry into force of China’s environmental protection tax law increases the carbon-related risks faced by businesses. This could potentially discourage the utilization of carbon-intensive technologies, encourage a voluntary shift toward more carbon-efficient technologies, and motivate enterprises to actively transition toward greener practices (29). Simultaneously, enterprises will be compelled by the escalating carbon risk to redirect their scarce R&D resources toward the development of eco-friendly technology, aiming to acquire environmental credibility and facilitate the transition to green practices through R&D diversion (30). Considering all the aforementioned factors, we may reach the conclusion that companies with elevated carbon risks are inclined to undergo a shift toward sustainability. Therefore, we put forward the subsequent conjecture:

*H*1: Carbon risk positively promotes the green transition of enterprises.

### 2.2 Paths mechanisms of carbon risk on green transition

In terms of Porter Hypothesis, moderate environmental constraint is conductive to induce firms to implement green technology innovation. This can lead to an innovation compensation effect that outweighs the expenditure of environmental policy compliance (14). Companies that implement eco-friendly innovations in their production and operations can overcome the entrenched reliance on high-carbon practices and reduce the expenses associated with environmental monitoring (31). According to the Porter Hypothesis, the remarkable benefits of eco-friendly innovation motivate leaders to adopt a green transition as their primary business strategy. The driving forces of corporate green transition come from internal and external sources, respectively. From the internal perspective, the main burden of the green transition lies on capital formation, accumulation, and transfers facilitated by full R&D investment in the green sector’s productivity (32). From the external perspective, based on institutional theory, Yang and Chi argue that green transition is a strategic choice for the purpose of obtaining legitimacy; According to stakeholder theory, the need to adhere to stakeholder green demands if a firm seeks to attain superior competitive positions and sustained performance (21). Based on the above analysis, companies facing internal and external pressures have a higher probability of generating sustainable green value and gaining a competitive edge over their rivals. Carbon risk impacts the green transition by influencing both the internal production structure optimization and the external stakeholders’ pressure. To begin with, in the internal R&D transformation, carbon risk propels R&D transition of enterprises, boosts the number of R&D staff, enhances the overall level of highly skilled individuals, and reallocates scarce R&D resources toward green technology advancement, consequently, gear up green transition of firms. Furthermore, stakeholder theorists have argued that stakeholder pressures can prompt a firm’s motivation to carry out green practices (21, 33). While carbon risk increases, it attracts greater focus from stakeholders which include analysts and institutional investors, stakeholders’ demands for environmental issues will bring great pressure to enterprises, the pressure is likely to promote corporate more transparent governance and higher commitment to ESG. As a company facing significant stakeholder pressure from stakeholder, its higher commitment to ESG will propel enterprises to actively exploit low-carbon technologies and carry out green transition initiatives, ultimately shaping a positive environmental reputation. Thus, this document puts forward the subsequent conjecture:

*H*2: The green transition is influenced by carbon risk through the internal R&D transformation and the pressures from external stakeholders.

### 2.3 Decomposition mechanism of green transition

Considering the theory of organizational learning, the exploration mainly composed of searching, adventure, experiment, innovation, and primarily concentrates on the invention and creation of new things. Exploitation consists of improvement, selection, production, implementation, etc., with more emphasis on the improvement of existing things (34). Given the administrative characteristics of green technology patent in China (35), we categorize green patents into invention green patents and utility green patents. The first one emphasizes innovation and creation, whereas the second one emphasizes enhancement and progress. The green transition in terms of invention and utility green patents is redefined as exploratory green transition (GPI) and exploitative green transition (GPA) (36, 37). The primary focus of the exploratory green transition is to innovate and develop advanced green and low-carbon technologies. This involves venturing into uncharted territory with longer time frames and higher levels of uncertainty regarding potential outcomes. The exploitative green transition primarily involves enhancing and advancing current green technologies and production processes, along with optimizing and utilizing existing technologies. It can achieve short-term results and relatively stable expected return. The green transition is categorized as either autonomous green transition (IGP) or cooperative green transition (UGP), depending on whether the green patent is filed independently or jointly. According to the above analysis, we formulate the subsequent hypotheses:

*H*3: The green transition of exploratory, exploitative, autonomous, and cooperative types is positively influenced by carbon risk.

**Figure 1:**
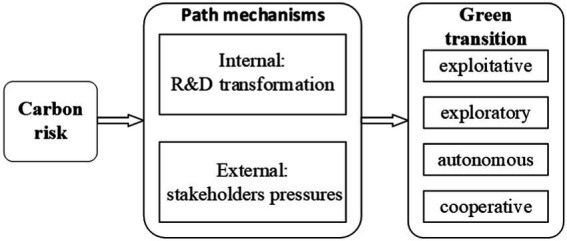
Theoretical framework.

Figure 1 shows the theoretical framework of this study.

## 3 Methodology and identification strategy

### 3.1 Samples and data collection

We describe the process of data collection in this section. The first dataset is green patents data, obtained from China Research Data Service Platform (CNRDS). The second dataset is carbon emissions data, collected from China Industrial Economic Statistical Yearbook and China Energy Statistical Yearbook. To test the relationship between carbon risk and corporate green transition, we use two measures of carbon risk. The first carbon risk measure is carbon emission amount, measured by the CO2 equivalent emissions in tons. The second measure is carbon intensity, measured as the ratio of CO2 emissions scaled by a firm’s operating cost. The third dataset includes control variables collected from CSMAR and WIND databases. Considering that Chinese manufacturing enterprises began to pay attention to environmental issues and carry out green transformation practices since 2010, and taking into account the availability of data, we select listed companies in China’s manufactural industry from 2011 to 2020 as the initial sample set. To address potential biases, The initial samples are treated as follows: (1) Eliminate the samples of IPO year, listing ST and delisted companies during the investigation period; (2) Wipe out the samples with missing key variable data and abnormal profit rate and asset-liability ratio; (3) In order to avoid the influence of potential outliers, we winsorize all continuous variables at the 1st and 99th percentiles. In the end, a total of 12,939 “firm-year” observation samples were obtained from 30 manufacturing sub-industries and 2,163 listed companies.

### 3.2 Empirical model

Following prior studies (8, 38–41), we probe the causal correlation between carbon risk and the green transition using the following regression model:


(1) $$\begin{array}{c} GP_{i,t}=\alpha_{0}+\alpha_{1}\times CR_{i,t}+\sum \mathrm{Control}s_{i,t}\\ +\sum \mathrm{Industr}y_{k}+\sum Year_{t}+\varepsilon_{i,t} \end{array}$$

Firms *i,* years *t,* industries *k*, and random interference terms *ε* are involved in the equation. The measured variable *GP_i,t_* represents the extent of green transformation of *i* firm during the *t* time frame, evaluated through variables related to green patent applications. CR*_i,t_* is the carbon risk of *i* firms in *t* period. ΣControls*_i,t_* are related control variables, ΣIndustry*_k_* is the fixed effect of industry, ΣYear*_t_* is the year-fixed effect. Heteroscedasticity is used to adjust standard errors. α_1_ is the regression coefficient we care about, and we predict it to be significantly positive. In order to alleviate the potential heteroscedasticity and sequence-related problems, standard errors of regression coefficients are adjusted by means of clustering at the firm level.

### 3.3 Variables

#### 3.3.1 Dependent variable: green transition

According to the existing research, the number of enterprise patents is a commonly used indicator to measure innovation performance (42),this paper uses the number of enterprises’ green patent applications as an indicator to measure green transition, and the number of green patent applications plus 1 to take the natural logarithm, and the log is utilized as the proxy variable for gaging green transition.

#### 3.3.2 Independent variable: carbon risk

Based on existing literature, carbon intensity is taken as the proxy variable of enterprise carbon risk (4, 8, 43). The advantage of using this proxy is not only its ability to represent the connotation and significance of carbon risk, but also better data availability. Carbon intensity is measured by dividing carbon emissions by operating income (6, 44). The precise calculation formula for carbon risk at the corporate level is as stated below.


(2) $$CR_{k,i,t}=\left(COC_{k,i,t}/\mathrm{IO}C_{k,t}\right)\times ICE_{k,t}/SR_{k,i,t}$$

CR*_k,i,t_* is the carbon risk of firm *i*, in *t* year and *k* industry, COC*_k,i,t_* presents the operating cost for firm *i*, in year *t* and in industry *k* based on CSMAR database. IOC*_k,t_* is the total operating cost of *k*-industry in year *t* based on WIND database, ICE is the total carbon emission of *k*-industry in year *t*, and its calculation formula is as follows: ICE = (total energy consumption of industry) × (carbon conversion coefficient). SR is the sales revenue of firms based on CSMAR database. The carbon conversion coefficient used is based on the carbon calculation standard of Xiamen Energy Conservation Center, where 1 ton of standard coal has a carbon conversion coefficient of 2.493. Equation (2) thus gives a quantifiable firm-specific carbon risk exposure.

#### 3.3.3 Control variables

To account for the potential impact of factors outside of this model, we controlled for the variables that may influence the green transition of firms. For instance, firms that are lager may have more resources to carry out green transition (45), so firm scale is controlled. Similarly, older firms may have had longer exposure to isomorphic processes related to green transition (46), so firm age is controlled. Along with the same logic, following previous studies on the green transition of corporate (33, 45), the control variables of our study include: firm scale (staff), age of listing (age), ratio of fixed assets (tang), asset-liability ratio (lev), sustainable growth rate (grow), profitability (ebit), operating cash flow (ocf), capital intensity (density), market value (Q), ownership concentration (top1), board size (board), duality (dual) and state-owned enterprises (soe). Given the skewed data, the logarithms of some variable values were taken. The detailed variable definitions are displayed in Table 1.

**Table 1:** Main variable definition.

Variable type	Variable name	Abbreviation	Definition
Dependent variable	Green patent	GP	The amount of green patent applications of enterprises plus 1 takes the logarithm
Independent variable	Carbon risk	CR	CO_2_ emissions/operating costs of enterprises
Control variables	Enterprise scale	staff	The number of employees takes the natural logarithm
	Age of listing	age	The time to list of enterprises takes natural logarithm
	Ratio of fixed assets	tang	Fixed assets/total book assets of firms
	Asset-liability ratio	lev	Total liabilities/total book assets of enterprises
	Sustainable growth rate	grow	Sales growth rate without changing operating efficiency and financial policies
	Profitability	ebit	Total earnings before interest and tax/operating income of enterprises
	Operating cash flow	ocf	Net cash flow/total book assets generated from business activities of enterprises
	Capital intensity	density	Total fixed assets/number of employees
	Market value	Q	Tobin Q, Enterprise Market Value/Total Book Assets
	Ownership concentration	top1	Shareholding rate of the largest shareholder of the enterprise
	Board size	board	The number of directors at the end of the period takes natural logarithm
	Duality	dual	For dummy variable, the value of chairman and general manager is 1, otherwise, it is 0
	State-owned enterprises	soe	Dummy variable, the value of state-owned enterprises is 1, otherwise the it is 0

## 4 Empirical results

### 4.1 Descriptive statistics and correlation analysis

Descriptive statistics of the main variables from 2011 to 2020 are shown in Column A of Table 2. It reveals that the mean value of green transition (GP) is 1.3784, the median value is 1.0986, and the standard deviation is 1.4861. The average value is higher than the median value, suggesting significant variations in the extent of green transition among manufacturing companies. The average carbon risk is 0.4444, which implies that firms in the sample emit 0.4444 kg of carbon per 1 China Yuan of sales. The median value of carbon risk is 0.1497, which indicates that the carbon risk of most sample firms is relatively low. The minimum value of carbon risk is 0.0417, and the maximum value of carbon risk is 2.3507, which demonstrates that carbon risk varies significantly among different enterprises. The values of other control variables are within a reasonable range, which are generally consistent with the values of previous studies. In Column B of Table 2, according to the median value of carbon risk, the samples are divided into lower carbon risk group and higher carbon risk group. The findings from the average T test and the median Wilcoxon Z test show that firms with higher carbon risk have superior level of green transition, and significant statistical differences preliminarily confirm the research hypothesis H1.

**Table 2:** Descriptive statistics of main variables.

Panel A: Descriptive statistics of main variables								
VarName	Obs	Mean	SD	Min	P25	Median	P75	Max
GP	12,939	1.3784	1.4861	0.0000	0.0000	1.0986	2.3979	5.4723
CR	12,939	0.4444	0.5785	0.0417	0.0924	0.1497	0.4696	2.3507
staff	12,939	7.7992	1.1091	5.4931	7.0166	7.7169	8.5080	10.7500
age	12,939	2.0352	0.7716	0.6931	1.3863	2.0794	2.7081	3.2581
tang	12,939	0.2290	0.1307	0.0214	0.1301	0.2054	0.3051	0.6030
lev	12,939	0.3816	0.1828	0.0534	0.2347	0.3728	0.5165	0.8036
grow	12,939	0.0648	0.0618	−0.0436	0.0228	0.0509	0.0895	0.3301
ebit	12,939	0.1225	0.0904	0.0094	0.0572	0.1004	0.1619	0.4650
ocf	12,939	0.0557	0.0625	−0.1097	0.0166	0.0525	0.0931	0.2339
density	12,939	1.9876	1.0645	0.4563	1.2822	1.7494	2.4158	6.6124
Q	12,939	2.0676	1.2057	0.8847	1.2964	1.6778	2.3902	7.6868
top1	12,939	0.3475	0.1388	0.1004	0.2400	0.3309	0.4356	0.7313
board	12,939	2.1212	0.1838	1.6094	1.9459	2.1972	2.1972	2.5649
dual	12,939	0.3064	0.4610	0.0000	0.0000	0.0000	1.0000	1.0000
soe	12,939	0.0819	0.2743	0.0000	0.0000	0.0000	0.0000	1.0000
Panel Descriptive statistics grouped by carbon risk								
Variable	Low CR		High CR		Mean diff	T test	P50 diff	Z test
	(1)	(2)	(3)	(4)	(3)–(1)		(4)–(2)	
	Mean	P50	Mean	P50				
GP	1.1396	1.0986	1.6173	1.6094	0.4777***	18.5223	0.5108***	16.3292

*, ** and *** indicate significance at the 10, 5 and 1% levels, respectively.

### 4.2 Baseline regression

To accurately explore the influence of carbon risk on the green transition, the benchmark regression test employs the model (1), and the findings are displayed in Table 3. Column (1) displays the influence of carbon risk (CR) on green transition (GP) without adding control variables. As seen from the table that the carbon risk coefficient (CR) exhibits a highly positive significance at the level of 1%, which reveals that carbon risk significantly promotes the green transition of enterprises with fixed effects for both year and industry. Column (2) report the effect of carbon risk (CR) on the green transition (GP) after controlling for other variables. The explanation of the model is further enhanced and still shows that carbon risk significantly promotes the green transition of firms, the hypothesis H1 is proved. Particularly, the coefficient of regression (0.2083) in column (2) holds significant economic importance. It suggests that a rise of one-standard-deviation (0.5785) in carbon risk results in an average increase 0.1205 (0.5785 × 0.2083) in the green transition. This increase is equivalent to 8.74% (0.1205/1.3784) of the dependent variable sample mean in terms of economic significance.

**Table 3:** The impact of carbon risk on the green transition.

	(1)	(2)
	GP	GP
CR	0.3416***	0.2083***
	(4.2408)	(2.7592)
staff		0.5651***
		(40.0829)
age		0.0615***
		(3.6691)
tang		−1.3849***
		(−14.6724)
lev		0.4929***
		(6.4135)
grow		1.4252***
		(5.8919)
ebit		−0.3840**
		(−2.0189)
ocf		0.1712
		(0.8326)
density		0.1222***
		(8.8311)
Q		−0.0210**
		(−2.0080)
top1		−0.1160
		(−1.4376)
board		0.2262***
		(3.6063)
dual		0.0022
		(0.0952)
soe		0.1308***
		(3.0664)
cons	1.2266***	−3.8227***
	(32.8868)	(−22.8452)
Controls	NO	YES
Year FE	YES	YES
Industry FE	YES	YES
R2_within	0.0013	0.2437
N	12,939	12,939

Table 3 reports the OLS regression results of the effect of carbon risk on the green transition. The t-value adjusted (reported in parentheses) are based on aggregated robust standard errors by firms. *, ** and *** indicate significance at the 10, 5 and 1% levels, respectively.

### 4.3 Endogeneity check

#### 4.3.1 2SLS instrumental variable method

The regression results demonstrate that firms with a high carbon risk are likely to carry out green transition. However, the relationship between carbon risk and green transition may have errors due to omitted variables and bidirectional causality. For instance, the corporate carbon risk may be related to unobservable factors such as industry, region and other potential factors. In addition, firms with a high degree of green transition may belong to high-carbon pollution industries, which generally have high carbon risks, and this potential bidirectional causality may produce certain endogeneity. We employ the heteroscedasticity instrumental variable to address potential endogeneity (47). The specific construction method of heteroscedasticity instrumental variable is as follow: the heteroscedasticity instrumental variable = (firm carbon risk value minus carbon risk mean value by industry and year as well as province)^3^ (expressed by IVCR). The rationale behind this is that the instrumental variable is associated with the current carbon risk, and it is unrelated to the current random error term. Specifically, the heteroscedasticity instrumental variable satisfy relevance and exclusivity criteria, thereby avoids weak instrumental variable problem.

Table 4 displays the two-stage regression outcomes of the heteroscedasticity instrumental variable. According to Column (1), the IVCR coefficient is positively significant at the 1% level, and the F statistic exceeds critical value of 10. It states clearly that the heteroscedasticity instrumental variable is highly related with current carbon risk and conforms with the correlation conditions. The second-stage estimation in column (2) still reflects that carbon risk significantly promotes the green transition of firms. These checks mitigate endogeneity concerns to a certain extent.

**Table 4:** Endogeneity and exclusivity tests.

	2SLS IV		Heckman two-step		Placebo test
	First stage	Second stage	First stage	Second stage	
	(1)	(2)	(3)	(4)	(5)
	CR	GP	DCR	GP	GP
CR		1.1497**		0.2108***	−0.0179
		(2.0894)		(2.7894)	(−0.9871)
IVCR	0.9810***				
	(9.6500)				
YPCR			0.2095**		
			(2.1674)		
IMR				−0.1841**	
				(−2.1485)	
cons	0.4740***	−3.6923***	0.6658***	−3.8404***	−3.7045***
	(8.6800)	(−9.4270)	(2.9247)	(−22.9363)	(−22.6761)
Controls	YES	YES	YES	YES	YES
Year FE	YES	YES	YES	YES	YES
Industry FE	YES	YES	YES	YES	YES
F/Wald	56.78	35343.58	2612.16	260.00	276.15
R2_within/Pseudo	0.0359	0.0920	0.2794	0.2439	0.2433
N	12,939	12,939	12,939	12,939	12,939

*, ** and *** indicate significance at the 10, 5 and 1% levels, respectively.

#### 4.3.2 Heckman two-stage model

The Heckman two-step approach is employed to investigate the possible issue of sample self-selection in hypothesis H1. In the initial step of the Probit model, the dummy variable DCR is set in terms of the median value of annual industry carbon risk. Additionally, the instrumental variable YPCR, representing the average value of yearly industry and provinces, is utilized. Using these variables, the regression outcomes enable the computation of the inverse Mills coefficient (IMR). During the second phase, IMR is utilized as a replacement for forecasting the value, and the outcomes that have been approximated are presented in Table 4. The positive regression coefficient of CR remains significant at the 1% level even after incorporating IMR and accounting for self-selection error, confirming the validity of the core conclusion.

#### 4.3.3 Placebo effect test

The above analysis confirms that carbon risk can propel the green transition of firms and consider potential endogenous problems such as omitted variables in samples and measurement errors. However, these results may simply be a placebo effect, given the limitations of factors that were not considered. Exclusive interpretation test was conducted by means of referring to the methods of previous studies (48). The carbon risk (CR) values of all sample data are extracted and randomly assigned to each observation value one by one for re-regression. If the placebo effect exists, the carbon risk (CR) after treatment is still positively correlated with the green transition. The carbon risk (CR) coefficient in column (5) of Table 4 is not significant, which is significantly different from the baseline regression results, from above analysis, we can exclude the existence of placebo effect.

#### 4.3.4 Propensity score matching

Considering possible distinction of initial conditions of firms with different carbon risk levels, we adopt a propensity score matching approach to alleviate potential endogeneity due to possible sample self-selection. The samples of firm-year observations were categorized into a treatment group and a control group. Firm-year observations that exceed the industry mean are included in the treatment group, on the contrary, the control group consisted of those with a carbon risk lower than the industry average. We employ annual matching to avoid sample selection bias arising from matching across years. To ensure that the two groups of samples have similar observable firm characteristics, we conduct the matching based on firm scale (staff), listing age (age), asset-liability ratio (lev), sustainable growth rate (grow), market value (Q), ownership concentration (top1), and R&D intensity (RDI) with a radius of 0.01. To assess the dependability of the PSM approach, one can observe the absolute value of the standard deviation of the variables that have been matched. A smaller absolute value of standard deviation leads to a more favorable matching effect. After matched, the equilibrium test results indicate that the standardized deviations of both the treatment group and the control group are substantially reduced, in terms of absolute value, they are all less than 5% in the post-match sample. Additionally, the T test results demonstrate that there are no significant differences among the characteristic variables post-match, which indicates that the results are robust after PSM matching.

Panel A in Table 5 demonstrates the test results of average treatment effects (ATT) of the treatment group in the pre- and post-match samples. We find that the differences are statistically significant, implying that the positive relationship between carbon risk and the green transition supports hypothesis H1. Next, we perform a regression analysis based on the matched sample using Equation (1), and the corresponding regression outcomes are presented in Panel B. Table 5 clearly states that the CR regression coefficient is highly positive at a significance of 1% level, both with and without control variables, which indicates that firms with a high carbon risk are more likely to carry out green transition. This finding aligns with the previous outcomes. All the results suggest that our main effects are robust.

**Table 5:** Propensity scores matching approach.

Panel A: Average treatment effect of the treated group						
Variable	Sample	Treated	Controls	Difference	S.E.	T-stat
GP	Unmatched	1.4750	1.2796	0.1954	0.0261	7.50***
	Matched	1.4755	1.4092	0.0663	0.0329	2.02**
Panel B: Regression based on PSM						
		(1)		(2)		
		GP		GP		
CR		0.3366***		0.2091***		
		(4.1566)		(2.7550)		
cons		1.2298***		−3.8363***		
		(32.7929)		(−22.8332)		
Controls		NO		YES		
Year FE		YES		YES		
Industry FE		YES		YES		
R2_within		0.0012		0.2437		
N		12,901		12,901		

*, ** and *** indicate significance at the 10, 5 and 1% levels, respectively.

#### 4.3.5 Exogenous shock test

##### 4.3.5.1 Model and regression analysis

To assess whether carbon risk promotes the green transition more steadily, we adopt the official entry into force of Environmental Protection Tax Law of the People’s Republic of China on January 1, 2018, as an exogenous shock, and evaluate the impact of the policy by means of difference-in-difference (DID) method. DID model can avoid the two-way causal problem to a great extent and can well eliminate the interference of other factors except policies. In addition, the model can alleviate omitted variables bias by means of time- and industry- fixed effect. The regression model is constructed as follows:


(3) $$\begin{array}{c} GP_{i,t}=\beta_{0}+\beta_{1}\times CR_{i,t}+\beta_{2}\times \mathrm{Treat}+\beta_{3}\times \mathrm{Post}+\beta_{4}\times \mathrm{Treat}\\ \times \mathrm{Post}+\sum \mathrm{Control}s_{i,t}+\sum \mathrm{Industr}y_{k}+\sum Year_{t}+\varepsilon_{i,t} \end{array}$$

The enterprise is represented by *i*, the year is represented by *t*, the industry is represented by *k*, and the random error term is represented by *ε*. Treat serves as a fictitious treatment variable, signifying its association with a treatment group. The carbon-intensive enterprises (treatment group) have a value of 1, while the other enterprises (control group) have a value of 0. The post serves as a placeholder for time, while the year of implementation of the Environmental Protection Tax Law in the People’s Republic of China marks the year of significant policy change. The value of Post is 1 in 2018 or later, otherwise it is 0. This paper identifies the carbon-intensive industries based on the carbon emissions of the manufacturing sub-industries in which the enterprise is situated. Regarding the industry’s carbon emission and energy consumption level, the carbon-intensive enterprises encompass those in six sub-industries: paper making, petrochemical, chemical industry, non-metallic mineral products, ferrous metals, and non-ferrous metals. On the other hand, the remaining enterprises are classified as non-carbon-intensive. The control variables, denotes as ΣControls*_i,t_*, are variables that are related to control conditions; ΣIndustry*_k_* represents the industry-fixed effect; ΣYear*_t_* represents the year-fixed effect.

The core coefficient in Equation (3) is *β_4_*. Our prediction is that the β4 will have a positive and statistically significant outcome. The results of the regression are shown in Table 6. The first column displays the regression outcome without control variables and without controlling year- and industry-fixed effects. The second column shows the corresponding outcome without control variables but with the inclusion of year- and industry-fixed effects. In the third column, the regression result includes control variables but does not account for year- and industry-fixed effects. Finally, the fourth column presents the regression result with both control variables and the simultaneous control of year- and industry-fixed effects. The regression coefficient(β4) of the interaction term (Treat*Post) in Table 6, specifically in columns (1)–(4), exhibit significant positive values of 0.2593, 0.1836, 0.2858, and 0.2289, respectively, at a significance of 1% level. The outcomes suggest that regardless of the inclusion of control variables or the control of year-and industry-fixed effects, the environmental sustainability of businesses will be remarkable enhanced by corporate carbon risk, thus confirming research hypothesis H1.

**Table 6:** DID regression.

	(1)	(2)	(3)	(4)
	GP	GP	GP	GP
Treat × Post	0.2593***	0.1836***	0.2858***	0.2289***
	(6.0554)	(4.3582)	(6.8819)	(5.4964)
Post	0.4300***	0.8287***	0.2137***	0.5157***
	(21.8017)	(21.7363)	(9.8120)	(11.7911)
Treat	0.3968***	0.4197**	0.3724***	0.5205***
	(4.5446)	(2.1026)	(4.6082)	(2.9565)
CR	−0.4027***	−0.0087	−0.3405***	−0.0813
	(−7.3830)	(−0.1256)	(−6.5114)	(−1.1741)
staff			0.5344***	0.5168***
			(28.0833)	(28.2603)
age			0.1621***	0.0725***
			(6.9317)	(3.0380)
tang			−1.1501***	−0.8129***
			(−10.1339)	(−7.3283)
lev			0.1694*	0.1611*
			(1.8956)	(1.8590)
grow			0.6282***	0.6465***
			(2.9137)	(3.0645)
ebit			−0.4685**	−0.2981
			(−2.5002)	(−1.6263)
ocf			−0.2464	−0.0562
			(−1.4879)	(−0.3430)
density			0.0972***	0.0648***
			(6.8306)	(4.6269)
Q			−0.0096	−0.0211**
			(−1.0440)	(−2.1381)
top1			−0.6395***	−0.2773**
			(−5.3292)	(−2.4315)
board			−0.0795	0.1159
			(−1.0604)	(1.5994)
dual			0.0141	−0.0029
			(0.5311)	(−0.1140)
soe			0.1004**	0.0480
			(2.2407)	(1.0939)
cons	1.1451***	0.1722	−2.7246***	−3.9188***
	(35.9393)	(1.1179)	(−12.9367)	(−16.1980)
Controls	NO	NO	YES	YES
Year FE	NO	YES	NO	YES
Industry FE	NO	YES	NO	YES
R2_within	0.0764	0.1471	0.1440	0.1739
N	12,939	12,939	12,939	12,939

*, ** and *** indicate significance at the 10, 5 and 1% levels, respectively.

##### 4.3.5.2 Parallel trend test

**Figure 2:**
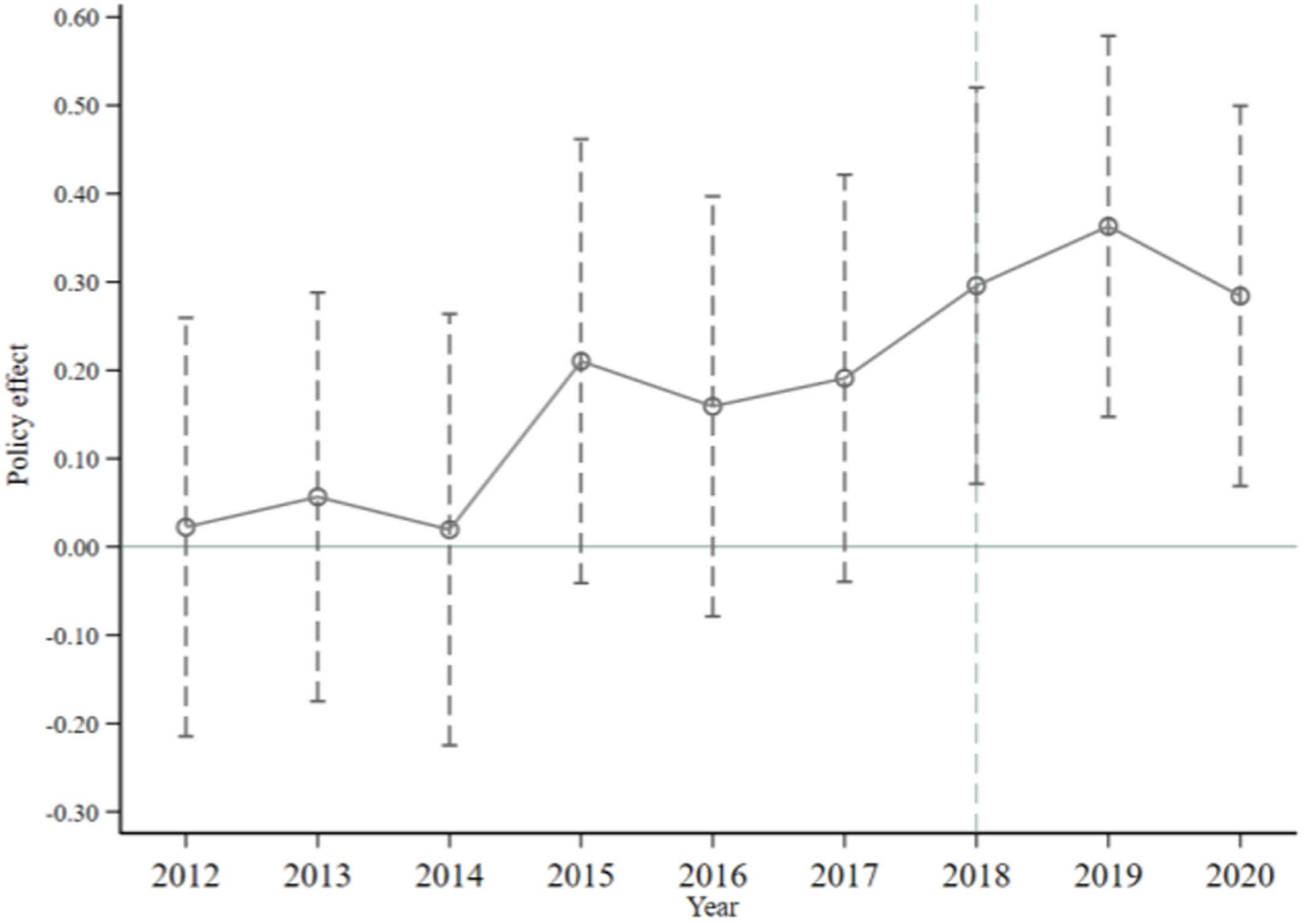
Parallel trend test.

To ensure the accuracy of DID regression model, it is requisite for both the treatment group and the control group to exhibit similar trends prior to exogenous policy shocks. This implies that the DID model should adhere to the hypothesis of parallel trend. The insignificance of the interaction coefficient can be observed in Figure 2 prior to the implementation of the exogenous policy. This suggests that there is no notable distinction between the treatment group and the control group prior to 2018, and the model aligns with the hypothesis of parallel trend. Following the implementation of the policy, the coefficient of interaction shows a notable positive trend, suggesting that the policy has greatly facilitated the green transformation of firms.

### 4.4 Robust check

#### 4.4.1 Alternative explained variable

The proxy variable for re-measuring the degree of sustainable transition is obtained by taking the logarithm of the count of green patents granted and adding 1. Table 7 displays the projected outcomes in columns (1) and (2). The CR’s regression coefficient is markedly positive at the level of 1%, both without and with control variables. The outcomes stay strong using an alternative dependent variable.

**Table 7:** Alternative variables regression.

	(1)	(2)	(3)	(4)
	AGP	AGP	GP	GP
CR	0.4500***	0.3012***		
	(6.2876)	(4.5145)		
WCR			0.4509***	0.2933*
			(2.8160)	(1.9495)
cons	0.8947***	−3.4956***	1.2846***	−3.7877***
	(27.1446)	(−23.3261)	(36.5993)	(−22.6710)
Controls	NO	YES	NO	YES
Year FE	YES	YES	YES	YES
Industry FE	YES	YES	YES	YES
R2_within	0.0029	0.2250	0.0006	0.2434
N	12,939	12,939	12,939	12,939

*, ** and *** indicate significance at the 10, 5 and 1% levels, respectively.

#### 4.4.2 Alternative core independent variable

The carbon emissions of different industries and different energy forms are classified and weighted, the carbon risk level of enterprises is re-calculated (8), and the carbon risk variable is replaced by WCR (Weighting CR). Table 7 displays the projected outcomes in columns (3) and (4). By including control variables, the CR regression coefficient exhibits a statistically markedly positive at the level of 10%. The conclusion is consistent with the above results by replacing the core independent variables.

#### 4.4.3 Multi-dimensional control of omitted variables

First, in view of the influence of industry, province and government subsidies and other aspects on the green transition of enterprises, we re-estimate model (1) based on the above-mentioned variables. Secondly, considering that the bidirectional fixed model of year and industry is not strict enough to control endogeneity, the high-order joint fixed effect method of controlling “Year*Province” is adopted for testing. Table 8 displays the projected outcomes in columns (1)–(3).

**Table 8:** Robustness test.

	(1)	(2)	(3)	(4)	(5)	(6)
	Subs	Indus/Prov	Year*Prov	Balance panel	Sub samples	Lag1
CR	0.1847**	0.1468**	0.1523**	0.5100***	0.2324***	
	(2.4440)	(1.9711)	(2.0175)	(3.1083)	(3.0080)	
L.CR						0.1722**
						(2.0977)
cons	−3.8522***	−3.8578***	−3.8109***	−3.5213***	−3.8469***	−3.8390***
	(−23.1385)	(−23.1811)	(−22.5111)	(−10.2268)	(−22.3097)	(−19.7992)
Controls	YES	YES	YES	YES	YES	YES
Year FE	YES	YES	YES	YES	YES	YES
Industry FE	YES	YES	YES	YES	YES	YES
R2_within	0.2482	0.2435	0.2408	0.2346	0.2397	0.2485
N	12,939	12,939	12,939	3,570	11,894	9,961

*, ** and *** indicate significance at the 10, 5 and 1% levels, respectively.

#### 4.4.4 Subsample regression

First, considering the impact of self-selection bias caused by enterprises entry and exit on green transition, the balanced panel data is retained for inspection. Secondly, eliminate the samples containing the words “environment,” “environmental protection,” “green” and “ecology” in the enterprise name, and at the same time eliminate the samples involving “environment,” “environmental protection,” “green” and “ecology” in the business scope of the enterprise. Table 8 displays the projected outcomes in columns (4) and (5).

#### 4.4.5 Lag one phase

To alleviate the possible endogeneity of bidirectional causality in regression, the core explanatory variables are treated with one-stage lag, and the estimated results are indicated in column (6) of Table 8. Based on Table 8, it can be inferred that the fundamental finding of H1, which states that the promotion of enterprise carbon risk has a positive impact on its green transition, remains unchanged, indicating a strong and reliable conclusion.

## 5 Expansive research

### 5.1 Path mechanism test

The empirical findings confirm the beneficial impact of carbon risk on the transition toward sustainability. In order to further examine theoretical hypothesis and path mechanisms by which carbon risk affects the transition to green, we have constructed the model outlined below:


(4) $$\begin{array}{c} \mathrm{Pat}h_{i,t}=\gamma_{0}+\gamma_{1}CR_{i,t}+\sum \mathrm{Control}s_{i,t}+\sum \mathrm{Industr}y_{k}\\ +\sum Year_{t}+\varepsilon_{i,t} \end{array}$$

In the above Equation (4), Path represents the specific Path mechanism between carbon risk and green transition, which corresponds to internal R&D transformation and external stakeholder pressures, respectively. The internal R&D transformation is realized by means of improving R&D capitalization rate (RDCAP) and optimizing human capital of R&D. The R&D capitalization rate is measured by the ratio of capitalized R&D costs divided by overall R&D expenditures. Referring to the existing studies, the optimization of internal human capital can promote the sustainable growth of enterprises (49). The optimization of human capital is measured by the ratio of R&D staff (RDH) and the ratio of individuals with postgraduate degrees or higher (MD). According to stakeholder theory (25, 50–52), the behavior of a firm is significantly influenced by the demands and pressures from external stakeholders. The need to adhere to stakeholder demands if a firm seeks to attain superior competitive positions and sustained performance (21, 53, 54). In this paper, external stakeholder pressures are measured by investor attention (INVES) and institutional investor shareholding ratio (INS). The remaining variables are identical to those in Equation (1) and are not explicitly mentioned.

According to Table 9, the concrete path mechanisms are displayed. In column (1), the carbon risk regression coefficient is positively significant at the 5% level. This suggests that firms with higher carbon risk have higher R&D capitalization rates, which are likely to propel firms to implement R&D transformation. The carbon risks regression coefficients in columns (2)–(3) exhibit significant positive effects at the 1% level. Regression coefficients indicate that firms with higher carbon risk are more likely to increase the proportion of R&D personnel and the proportion of highly educated employees. The findings reveal that carbon risk promotes corporate green transition through R&D transformation which includes increasing R&D capitalization rate, increasing the proportion of R&D personnel and the proportion of highly educated employees. The regression coefficients of (4) and (5) are both statistically significant, which demonstrate that carbon risk can propel firms to carry out green transition through the pressures from external stakeholders. The results are consistent with previous studies (21, 24, 55–57). The hypothesis H2 is verified by the regression findings presented in Table 9.

**Table 9:** Path analysis.

	Internal: R&D transformation			External: Stakeholders pressures	
	(1)	(2)	(3)	(4)	(5)
	RDCAP	RDH	MD	INVES	INS
CR	0.0023**	0.0086***	0.0019***	0.1087*	0.0411***
	(2.1485)	(3.4646)	(2.9036)	(1.7387)	(3.2089)
cons	−0.0149**	0.0493***	−0.0209***	−2.7428***	−0.7845***
	(−2.4979)	(4.7580)	(−3.0367)	(−7.3020)	(−29.0729)
Controls	YES	YES	YES	YES	YES
Firm FE	NO	NO	YES	YES	NO
Year FE	YES	YES	YES	YES	YES
Industry FE	YES	YES	NO	NO	YES
R2_within	0.0209	0.0341	0.0758	0.1401	0.3485
N	12,939	12,939	12,939	12,939	12,939

*, ** and *** indicate significance at the 10, 5 and 1% levels, respectively.

### 5.2 Decomposition mechanism test

Furthermore, to examine the underlying mechanism of how carbon risk affects the shift toward sustainability, we categorize the transition into four types: exploratory green transition (GPI), exploitative transition (GPA), independent green transition (IGP), and cooperative green transition (UGP). Proxy variables such as the count of green invention patent applications, green utility patent applications, independent green patent applications, and joint green patent applications replace the explained variable (GP) in model (1). The findings presented in Table 10 suggest that the carbon risk associated with corporations positively influences exploratory, exploitative, independent, and cooperative green transitions. These results align with the findings and support hypothesis H3.

**Table 10:** Decomposition mechanism.

	Relation Type		Innovation Type	
	Autonomous	Cooperative	Exploration	Exploitation
	(1)	(2)	(3)	(4)
	IGP	UGP	GPI	GPA
CR	0.1418*	0.1868***	0.1127*	0.2708***
	(1.9302)	(4.0182)	(1.7464)	(4.2335)
cons	−3.4649***	−1.7987***	−3.3022***	−3.0715***
	(−20.9967)	(−15.6120)	(−22.0711)	(−21.0301)
Controls	YES	YES	YES	YES
Year FE	YES	YES	YES	YES
Industry FE	YES	YES	YES	YES
R2_within	0.2199	0.1083	0.2155	0.2130
N	12,939	12,939	12,939	12,939

*, ** and *** indicate significance at the 10, 5 and 1% levels, respectively.

The corporate carbon risk has a more remarkable impacts on promoting exploitative and cooperative green transition. This conclusion shows that the positive effects are not balanced. Against the background of carbon risk, enterprises are more inclined to carry out exploitative and cooperative green transition with low difficulty, high certainty, and fast short-term returns. Enterprises is likely to cooperate with other enterprises in order to carry out green transition and engage in incremental improvement of existing technologies or processes. The enthusiasm for exploratory and independent green transition with high uncertainty, great difficulty and no clear expected return in the short term is not high. The innovation of cutting-edge low-carbon technologies is insufficient, and the balance and quality of green transition need to be further improved.

### 5.3 Heterogeneity analysis

Based on analyzing internal mechanism of carbon risk affecting the green transition of firms, in view of the typical negative externalities of carbon risk, which are closely related to the characteristics of capital density, property rights, government subsidies and financing constraints, the following models is constructed to analyze the heterogeneous impacts of carbon risk as follows:

(5) $$\begin{array}{c} GP_{i,t}=\phi_{0}+\phi_{1}CR_{i,t}\times Grp_{i,t}+\phi_{2}CR_{i,t}+\phi 3Grp_{i,t}\\ +\sum \;\mathrm{Control}s_{i,t}+\sum \;\mathrm{Industr}y_{k}+\sum Year_{t}+\varepsilon_{i,t} \end{array}$$

In the above Equation (5), Grp corresponds to the grouping variables of capital density, government subsidies and financing constraints, respectively. If the value of the variable is higher than the median, assign 1; otherwise, assign 0. Where capital density is measured by dividing total assets by operating income. The determination of property rights is assessed based on whether it pertains to a government-owned establishment, with a value of 1 assigned to state-owned enterprises; otherwise, it is assigned a value of 0. The level of financing constraints is gaged using the KZ index. Other variables are the same as Equation (1).

Table 11 displays the heterogeneous effects of carbon risk on the density of enterprise capital, property rights, government subsidies, and financing constraints. The positive impacts of carbon risk on the green transition of enterprises are evident in columns (1)–(4) of Table 11, aligning with previous findings. In column (1), the interaction coefficient for capital density is markedly negative at the level of 1%. This indicates that compared with high capital-intensive enterprises, the carbon risk of low capital-intensive companies has more remarkable positive influence on promoting green transition. Regarding the essence of ownership rights, the interaction coefficient in column (2) exhibits a highly positive significance at the 1% level. This implies that the advancement of the green transition through carbon risk mitigation holds greater importance within state-owned enterprises. The coefficient of interaction in column (3) shows a remarkable negative effect at the 1% significance level. This suggests that, in comparison to enterprises receiving high government subsidies, low government subsidy enterprises exhibit a more pronounced promotion of green transition in terms of carbon risk. In column (4), the interaction coefficient is highly negative at a significance level of 1%, suggesting that the impact of carbon risk on green transition is more markedly for enterprises with low financing constraints compared to those with high financing constraints. According to the heterogeneity test findings, carbon risk has a greater impact on driving the transition toward sustainability in companies that have limited capital density, minimal government subsidies, few financing constraints, and are state-owned. The interaction coefficient in column (3) is markedly negative at the level of 1%, which indicates that compared with high government subsidy enterprises, the carbon risk of low government subsidy enterprises promotes green transition more obviously.

**Table 11:** Heterogeneity analysis.

	(1)	(2)	(4)	(5)
	Capd	Soe	Subs	KZ
CR*Grp	−0.1663***	0.1780***	−0.0939***	−0.1502***
	(−4.1887)	(2.7781)	(−2.6146)	(−3.7915)
CR	0.2607***	0.1953***	0.2566***	0.2688***
	(3.4370)	(2.5873)	(3.3031)	(3.4023)
Grp	0.1983***	0.0364	0.1904***	−0.0422
	(5.6279)	(0.6721)	(7.1208)	(−1.1219)
cons	−3.9089***	−3.8303***	−3.9065***	−3.8829***
	(−23.2581)	(−22.8913)	(−23.7806)	(−23.4799)
Controls	YES	YES	YES	YES
Year FE	YES	YES	YES	YES
Industry FE	YES	YES	YES	YES
R2_within	0.2457	0.2442	0.2469	0.2438
N	12,939	12,939	12,939	12,906

*, ** and *** indicate significance at the 10, 5 and 1% levels, respectively.

## 6 Conclusion

Derived from the data of Chinese manufacturing companies listed between 2011 and 2020. By utilizing industry energy consumption, we calculate the carbon risk data at the firm-level and thoroughly examine the influence of enterprise carbon risk on the transition toward sustainability. The conclusions mainly include: (1) Carbon risk at the firm level exhibits a strong positive correlation with the green transition. This transition is characterized by the overall advancement of exploratory, exploitative, autonomous, and collaborative efforts toward sustainability. The outcome remains consistent after undergoing a range of examinations. (2) The mechanism examination indicates that the enhancement of internal manufacturing framework and the consideration of external stakeholders are the pathways through which carbon risk affects the transition toward sustainability of a firm. (3) According to the analysis of heterogeneity, enterprises with low capital density, low government subsidies, low financing constraints, and state ownership are more influenced by carbon risk in driving the transition toward green practices. The robustness of the regression results using different indicators for green transition and carbon risk is evident. To tackle potential endogenous issues, we employ various techniques including instrumental variable approach, Heckman two-step method, placebo examination, PSM test, and DID measure. From a finer-granularity perspective, the research findings confirm the validity of the ‘Porter Hypothesis’ and contribute to the existing body of knowledge on carbon risk and the transition toward sustainability within individual companies.

### 6.1 Theoretical and practical implications

Our study is a strong foundation and crucial stepping-stone for further understanding the relationship between carbon risk and corporate green transition. The link between carbon risk perspective and green action is something that benefits both carbon neutrality and green transition literature. We believe that the joint consideration of internal R&D transformation and external stakeholder pressures provides a number of additional insights into why and how firms are motivated to carry out green transition in the context of achieving carbon neutrality. Our study also provides insights into the green transition of manufacturing firms in emerging economies. To begin with, companies ought to enhance their investment in eco-friendly innovation, increase the ratio of research and development staff and top-notch professionals, improve the overall R&D human capital level, optimize the internal production structure by means of R&D transformation. Secondly, firms should pay attention to the balance of green transition. To ensure the short-term survival and long-term sustainable development of businesses, it is crucial to enhance investment in both innovative and autonomous green transformation. This entails maintaining a harmonious equilibrium between exploratory and exploitative approaches, as well as independent and collaborative efforts toward green transition. Third, optimize the government’s environmental policy. Timely revisions to environmental regulation standards, implementation of rational fiscal and tax support measures, enhancement of government subsidy distribution methods, and augmentation of green finance assistance for advanced low-carbon technologies.

### 6.2 Research limitations and prospects

Even though the results of our paper overwhelmingly indicate that the relationship is strong positive between carbon risk and corporate green transition, there are still some limitations to this study. The single-industry, single-country limitations point to the need to investigate whether other industries and countries have the same experience. Due to the selection of listed manufacturing companies in China, the sample companies are all large enterprises, and the conclusions have certain limitations and may not be applicable to small and medium-sized enterprises. In the future, we can choose other emerging market scenarios or developed countries in Europe and the United States to explore the relationship between carbon risk and corporate green transition. The composite sample method can also be adopted to select cross-country samples or cross-scale enterprise samples, so as to make the conclusions more universal. As for the mechanism test between carbon risk and enterprise green transition, our study only explores the path mechanism of internal R&D transformation and the pressure of external institutional investors. Future research can explore other path mechanisms through which carbon risk affects corporate green transition.

## Data availability statement

The original contributions presented in the study are included in the article/supplementary materials, further inquiries can be directed to the corresponding author.

## Author contributions

JL: Data curation, Software, Writing – original draft, Formal analysis, Resources, Validation, Visualization, Writing – review & editing. YX: Conceptualization, Project administration, Supervision, Writing –original draft, Methodology. XG: Funding acquisition, Resources, Writing – review & editing, Formal analysis, Methodology, Validation. QW: Formal analysis, Methodology, Validation, Writing – review & editing.
